# Association Between Digoxin Use and Cancer Incidence: A Propensity Score-Matched Cohort Study With Competing Risk Analysis

**DOI:** 10.3389/fphar.2021.564097

**Published:** 2021-03-31

**Authors:** Chi-Jung Tai, Yi-Hsin Yang, Tzyy-Guey Tseng, Fang-Rong Chang, Hui-Chun Wang

**Affiliations:** ^1^ Graduate Institute of Natural Products, College of Pharmacy, Kaohsiung Medical University, Kaohsiung, Taiwan; ^2^ Department of Family Medicine, Pingtung Hospital, Ministry of Health and Welfare, Pingtung, Taiwan; ^3^ School of Pharmacy, College of Pharmacy, Kaohsiung Medical University, Kaohsiung, Taiwan; ^4^ National Institute of Cancer Research, National Health Research Institutes, Tainan, Taiwan; ^5^ Department of Family Medicine, Kaohsiung Medical University Hospital, Kaohsiung Medical University, Kaohsiung, Taiwan; ^6^ Department of Marine Biotechnology and Resources, National Sun Yat-sen University, Kaohsiung, Taiwan; ^7^ Drug Development and Value Creation Research Center, Kaohsiung Medical University, Kaohsiung, Taiwan; ^8^ Department of Medical Research, Kaohsiung Medical University Hospital, Kaohsiung Medical University, Kaohsiung, Taiwan

**Keywords:** β-blocker, competing risk analysis, cancer, digoxin, propensity-score matching

## Abstract

**Background:** Previous studies neglected death as a critical competing risk while estimating the cancer risk for digoxin users. Therefore, the current study aims to assess the effectiveness of digoxin on cancer prevention by competing risk analysis.

**Methods:** We performed a population-based retrospective cohort study using the Taiwan National Health Insurance Research database between 1998 and 2010. After one-to-one propensity score-matching from 36,160 patients with defined criteria, we enrolled 758 patients both in digoxin and β-blocker group for further analysis.

**Results:** The results showed that the digoxin group had higher all-cause mortality than the β-blocker group in the 4- year (10.4 vs. 4.9%) and 8 years (13.6 vs. 7.0%) follow-up. The subdistribution HR of cancer incidence in the digoxin group compared to the β-blocker group was 1.99 (95% confidence interval [CI]: 1.22–3.01) and 1.46 (95% CI: 1.01–2.15) in the 4 years and 8 years follow-up, respectively.

**Conclusions:** The result of our study showed the usage of digoxin has no benefit in cancer prevention compared with β-blocker. The possibility of β-blocker as a new drug candidate for cancer prevention needs further clinical evaluation. The current study also emphasized the necessity of competing risk analysis applying to similar clinical researches.

## Introduction

Although plants expressing cardiac glycosides have been used in medicine since ancient Egyptians times, digoxin, a potent inhibitor of Na^+^/K^+^-ATPase, was only first approved for the treatment of heart failure (HF) in 1998 by the United.States. Food and Drug Administration (Uretsky et al., 1993). Then, digoxin is currently used in patients with HF, atrial fibrillation (AF), and atrial flutter (AFL) (Sethi et al., 2018). However, since the development of novel drugs for HF, including β-blockers, angiotensin-converting-enzyme inhibitors (ACEIs), and angiotensin receptor blockers (ARBs), digoxin started to play a limited role in the treatment of patients with HF. Moreover, the prescriptions of digoxin have been declined gradually in patients with HF, AF or AFL because of its possible toxicity, narrow therapeutic range, and the potential for drug-drug interactions (Eichhorn and Gheorghiade, 2002; Xie et al., 2017; Sethi et al., 2018).

In addition to positive inotropic effects and the ability to decrease ventricular rates, repurposing digoxin as well as the development of other cardiac glycosides for cancer prevention and treatment has risen in highly discuss because of its anti-cancer properties *in vitro* (Prassas and Diamandis, 2008). There is no direct evidence of clinical trials to evaluate digoxin on cancer due to practical constraints. The available evidence predominantly based on observational studies but demonstrated controversial results. Digoxin was reported to increase the incidence rate of breast cancer using the database in Denmark, the United States, and the United Kingdom (Ahern et al., 2008; Biggar et al., 2013; Ahern et al., 2014; Couraud et al., 2014a). Moreover, digoxin use showed increased the risk of colorectal, lung, and uterine cancers analyzing the database in Denmark and the United Kingdom (Biggar et al., 2012; Boursi et al., 2014; Couraud et al., 2014b). On the contrary, studies from Sweden, Ireland, and Taiwan indicated that digoxin decreased the risk of prostate and liver cancers (Flahavan et al., 2014; Lim et al., 2015; Xie et al., 2017). The meta-analyses make a great effort to pool twenty-seven studies that showed digoxin increased the risk of estrogen receptor-positive breast cancer but not others (Osman et al., 2017); however, the heterogeneity of the previous studies might limit the data interpretation.

Probing deep into previous studies, we noticed several possible confounding factors that potentially contribute to the cancer risk associated with digoxin as follows. Firstly, some studies had confounding by indication, a phrase that refers to a situation where patient characteristics, rather than the intervention, are independent predictors of outcome (Cnossen et al., 2018). As a consequence, patients exposed and not exposed to digoxin might not be comparable, which hindered the relational inference (Biggar et al., 2012; Chung et al., 2017). Moreover, the previous studies neglected the possible cancer-preventive effect of concomitant medications, such as aspirin, ACEI, and ARB (Chung et al., 2017). Secondly, digoxin users are patients with HF, AF, or AFL, whose all-cause mortality is high and most of the previous studies neglected the competing risk of death. The bias associated with the competing risk of death happened in study design and methods for statistical analysis. For example, a case-control study design using specific cancer database was not appropriate because death might occur before cancer diagnoses (Ahern et al., 2008). Additionally, conventional approaches to describe the risk of cancer, including Kaplan-Meier (KM) survival analysis and Cox proportional hazards regression, can overestimate the risk of cancer by ignoring the competing risk of death (Haggstrom et al., 2016). These methods may lead to a biased result, especially when there is a big difference in mortality between the two groups (Ahern et al., 2014; Xie et al., 2017). Thirdly, medication adherence is a crucial factor associated with the incidence of cancer (Forbes et al., 2018). Specific medication has an impact on cancer incidence only when it was prescribed for chronic conditions, which need to be taken on a long-term basis. However, the medication adherence was not described in detail (Karasneh et al., 2015), or the exposure of digoxin was low in the previous studies (Boursi et al., 2014).

The following approaches in this study try to minimize the possible confounders and avoid violation of the assumption. Firstly, we evaluated the cancer incidence between digoxin and β-blockers in patients with HF, AF, or AFL. The indication of digoxin and β-blockers was almost identical in selected clinical conditions, which prevented confounding by indication (Kirchhof et al., 2016). Secondly, we designed a retrospective cohort study propensity score-matched with age, sex, medical comorbidities, and concomitant medications using a population-based National Health Insurance Research Database (NHIRD) in Taiwan. Finally, we calculated and matched the first-year cumulative defined daily dose (cDDD) of digoxin and β-blockers between two groups (Lin et al., 2016). The current study aimed to analyze the association between digoxin and cancer incidence through rigorous study design.

## Methods

### Data Source

The current study used one of the subsets of NHIRD, which contained two million patients (approximately 10% of Taiwan’s population) randomly selected from NHI beneficiaries in Taiwan. Because the prescriptions of digoxin have been declining steadily over the last 15 years, which may have caused selection bias (Goldberger and Alexander, 2014), we used a dataset from 1997 to 2010 to reduce the possible selection bias due to clinical tendency.

### Study Cohort and Design

**FIGURE 1:**
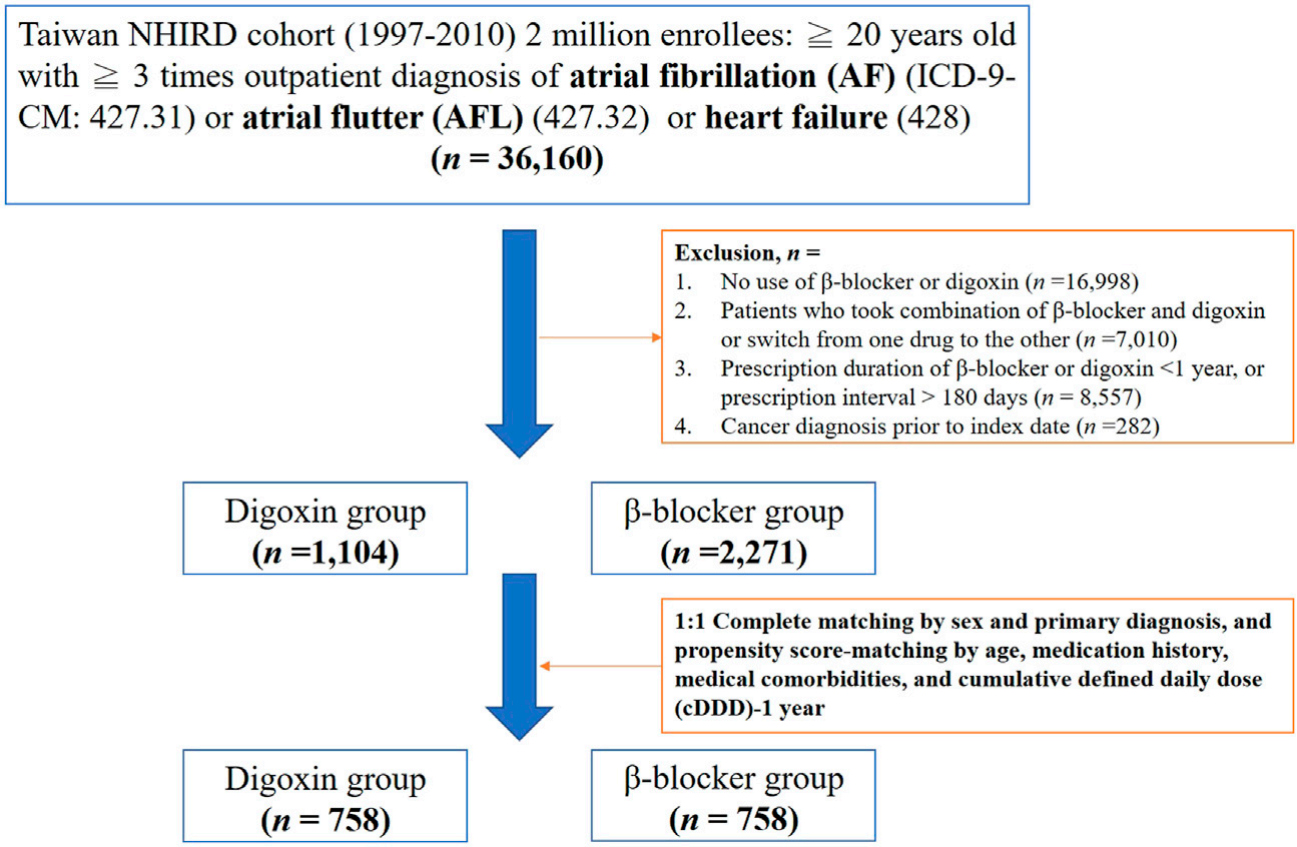
Study flow chart. Nhird, national health insurance research database.

**FIGURE 2:**
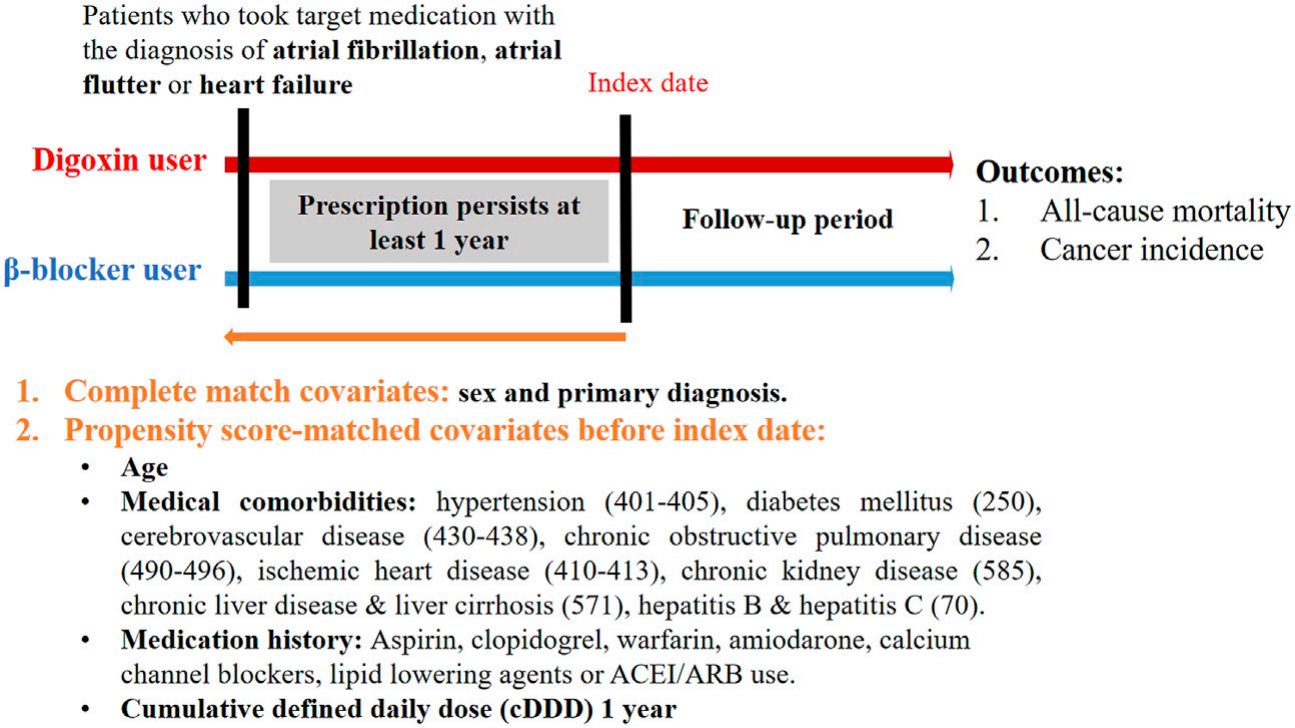
Study design.

We searched the records in NHIRD from 1998–2010 to identify patients aged ≥20 years with newly diagnosed HF, AF or AFL in our study. HF, AF, or AFL patients were identified by the International Classification of Diseases Revision, Ninth Revision, Clinical Modification (ICD-9-CM) codes, where 428 is for HF, 427.31 code is for AF, and 427.32 code is for AFL. To enhance diagnostic validity, patients with at least three consistent diagnoses of HF, AF, or AFL from outpatient medical records were enrolled in our study (Figure 1). We targeted β-blocker users as the control group because the clinical indication of digoxin and β-blockers was similar in patients with HF, AF or AFL (Kirchhof et al., 2016; Ponikowski et al., 2016). The prescriptions of digoxin and β-blockers were selected by Anatomical Therapeutic Chemical (ATC) classification system and the corresponding drug codes from outpatient medical claims using NHIRD. For at least a 1 year baseline prescription period, a year from the first prescription of target medications was assigned as the index date (Figure 2). To quantify individual’s exposure to target medications and medical adherence, the cDDD of target medications from the first prescription date to the index date (cDDD-1 year) were calculated (Tsai et al., 2016).

To enhance the validity of the current study, we made some exclusion criteria as follows (Figure 1): Firstly, patients without the use of digoxin and β-blockers in the treatment of HF, AF or AFL were excluded. Secondly, we excluded patients who have ever taken the combination of β-blockers and digoxin or switched from one drug to the other during the follow-up. Thirdly, drugs usually require long-term and sustained use to affect the incidence of cancer, so we excluded patients whose prescription duration of β-blockers and digoxin less than one year or prescription interval >180 days (Lam and Fresco, 2015). The process ensured medical adherence and persistence (Raebel et al., 2013). Finally, we excluded patients with cancer history prior to the index date (Figures 1,2).

To reduce demographic differences between the digoxin group and the β-blocker group, we performed propensity-score matching by age, sex, medical comorbidities, medication history and the cDDD of target medications (Lin et al., 2016). The medical comorbidities including hypertension, diabetes mellitus, cerebrovascular disease, chronic obstructive pulmonary disease, ischemic heart disease, chronic kidney disease, chronic liver disease or liver cirrhosis, and hepatitis B or C were selected by corresponding ICD-9-CM codes in outpatient medical claims previous to the index date (Figure 2). Medications such as aspirin, clopidogrel, warfarin, amiodarone, calcium channel blockers (CCBs), lipid-lowering agents, ACEI, and ARB were also selected as matching covariates, which were commonly prescribed to patients with HF, AF or AFL. Additionally, we matched the cDDD-1 year between the digoxin group and the β-blocker group, which represented that medication adherence and dose-response effect were similar in two groups (Raebel et al., 2013). The cDDD-1 year represented the cumulative dose of target medications during the first year.

The primary outcome of the current study was cancer incidence (ICD-9-CM: 140–208), which was identified from the inpatient medical records. As we mentioned above, death was a competing risk event because its occurrence cannot be treated as independent censoring when analyzing the time to cancer occurrence. Therefore, the in-hospital mortality was identified as a competing risk event from the inpatient medical records using NHIRD. The current study was approved by the Institutional Review Board of Antai Medical Care Cooperation Antai-Tian-Sheng Memorial Hospital in 2018.

### Statistical Analyses

Descriptive statistics were used to assess patients’ demographics. The standardized mean differences were calculated for the matched cohort to assess balance in measured baseline covariates (Austin, 2009). Some researchers had proposed that a standardized difference of more than 0.1 denoted meaningful imbalance in the baseline covariates (Normand et al., 2001). The adjusted HR of all-cause mortality and cancer incidence were calculated by Cox proportional hazard model adjusting for possible confounders. Importantly, the cumulative cancer incidence was estimated by the cumulative incidence competing risk method, which incorporated competing risks of death in the cumulative incidence function (Hsieh et al., 2015). The subdistribution HR of cancer incidence was performed by the proportional subdistribution hazards regression. All of the analyses were conducted using SAS version 9.4 (SAS Institute Inc., Cary, NC, United States), and SAS macros %CIF and %PSHREG were used for competing risk analyses (Lin and Johnston, 2012; Kohl et al., 2015).

## Results

### Baseline Characteristics

As shown in Figure 1, there were 36,160 newly diagnosed patients with HF, AF or AFL who met inclusion criteria. Following the application of exclusion criteria, we identified 1,104 patients in the digoxin group and 2,271 patients in the β-blocker group. Prior to propensity score matching, digoxin group patients were significantly older (71.8 ± 13.7 vs. 66.6 ± 13.4 years), and had a higher rate of warfarin usage (16.8 vs. 6.4%) and chronic obstructive pulmonary disease (COPD) (43.8 vs. 28.7%) compared to the β-blocker group (Table 1). Moreover, the cDDD-1 year was higher in the digoxin group than in the β-blocker group (183.2 ± 87.6 vs. 155.7 ± 122.2). In contrast, the β-blocker group more frequently used aspirin, clopidogrel, amiodarone, CCBs, lipid-lowering agents, and ACEI/ARB concomitantly. They also had higher prevalence of hypertension, diabetes mellitus, cerebrovascular disease (CVD), ischemic heart disease, chronic kidney disease (CKD), chronic liver disease, and hepatitis B or C (Table 1). These data suggested that the clinical demographics of patients taking digoxin and β-blocker were still quite different.

**TABLE 1:** Clinical demographics of patients before and after 1:1 propensity score-matching.

	Before matching		*p* Value	1: 1 matching		Standardized mean difference^a^
	Digoxin group *n* = 1104	β-blocker group *n* = 2271		Digoxin group *n* = 758	β-blocker group *n* = 758	
**Age (years)**	71.8 ± 13.7	66.6 ± 13.4	<0.001^b^	70.5 ± 14.4	70.2 ± 12.2	0.022
**Male**	610 (55.3%)	1212 (53.4%)	0.30	412 (54.4%)	412 (54.4%)	<0.001
**Medication history**						
Aspirin	526 (47.6%)	1295 (57.0%)	<0.001^c^	394 (52.0%)	378 (49.9%)	0.042
Clopidogrel	43 (3.9%)	303 (13.3%)	<0.001^c^	41 (5.4%)	48 (6.3%)	0.038
Warfarin	185 (16.8%)	146 (6.4%)	<0.001^c^	95 (12.5%)	91 (12.0%)	0.015
Amiodarone	74 (6.7%)	223 (9.8%)	0.003^c^	59 (7.8%)	64 (8.4%)	0.022
CCBs	430 (39.0%)	1293 (56.9%)	<0.001^c^	350 (46.2%)	361 (47.6%)	0.028
Lipid lowering agents	125 (11.3%)	672 (29.6%)	<0.001^c^	111 (14.6%)	123 (16.2%)	0.044
ACEI/ARB	656 (59.4%)	1472 (64.8%)	0.002^c^	467 (61.6%)	461 (60.8%)	0.016
**Medical comorbidities**						
Hypertension	663 (60.1%)	1853 (81.6%)	<0.001^c^	554 (73.1%)	550 (72.6%)	0.011
Diabetes mellitus	257 (23.2%)	624 (27.5%)	0.01^c^	197 (26.0%)	184 (24.3%)	0.039
Cerebrovascular disease	249 (22.6%)	447 (19.7%)	0.05	173 (22.8%)	162 (21.4%)	0.034
COPD	483 (43.8%)	651 (28.7%)	<0.001^c^	291 (38.4%)	302 (39.8%)	0.029
Ischemic heart disease	182 (16.5%)	663 (29.2%)	<0.001^c^	159 (21.0%)	150 (19.8%)	0.030
Chronic kidney disease	43 (3.9%)	148 (6.5%)	0.002^c^	36 (4.8%)	38 (5.0%)	0.009
Chronic liver disease	117 (10.6%)	391 (17.2%)	<0.001^c^	92 (12.1%)	96 (12.7%)	0.018
Chronic hepatitis B or C	150 (13.6%)	467 (20.6%)	<0.001^c^	121 (16.0%)	124 (16.4%)	0.011
**cDDD-1 year**	183.2 ± 87.6	155.7 ± 122.2	<0.001^b^	173.8 ± 82.8	177.4 ± 142.1	0.031

ACEI = angiotensin-converting enzyme inhibitor; ARB = angiotensin receptor blockers; CCBs = calcium channel blockers; COPD = chronic obstructive pulmonary disease; cDDD = cumulative defined daily doseLipid lowering agents include statin, fibrates, ezetimibe and niacin.

^a^ Standardized mean difference of more than 0.1 denotes meaningful imbalance in the baseline covariates.

^b^ Independent *t*-test: *p* -value <0.05.

^c^ chi-square test: *p* -value <0.05.

After 1:1 propensity-score matching by criteria, there were 758 patients in the digoxin group and 758 patients in the β-blocker group. The cancer-related covariates were well-balanced after matching (Table 1). The mean follow-up was 4.3 ± 3.1 years. The mean age of two groups was 70.5 ± 14.4 vs. 70.2 ± 12.2 years in the digoxin and the β-blocker group, respectively. There were more male patients (54.4 vs. 45.6%) in both groups. More than 50% of patients concurrently took aspirin or ACEI/ARB with target medications, and more than 70% of patients had a history of hypertension.

### Outcomes

**FIGURE 3:**
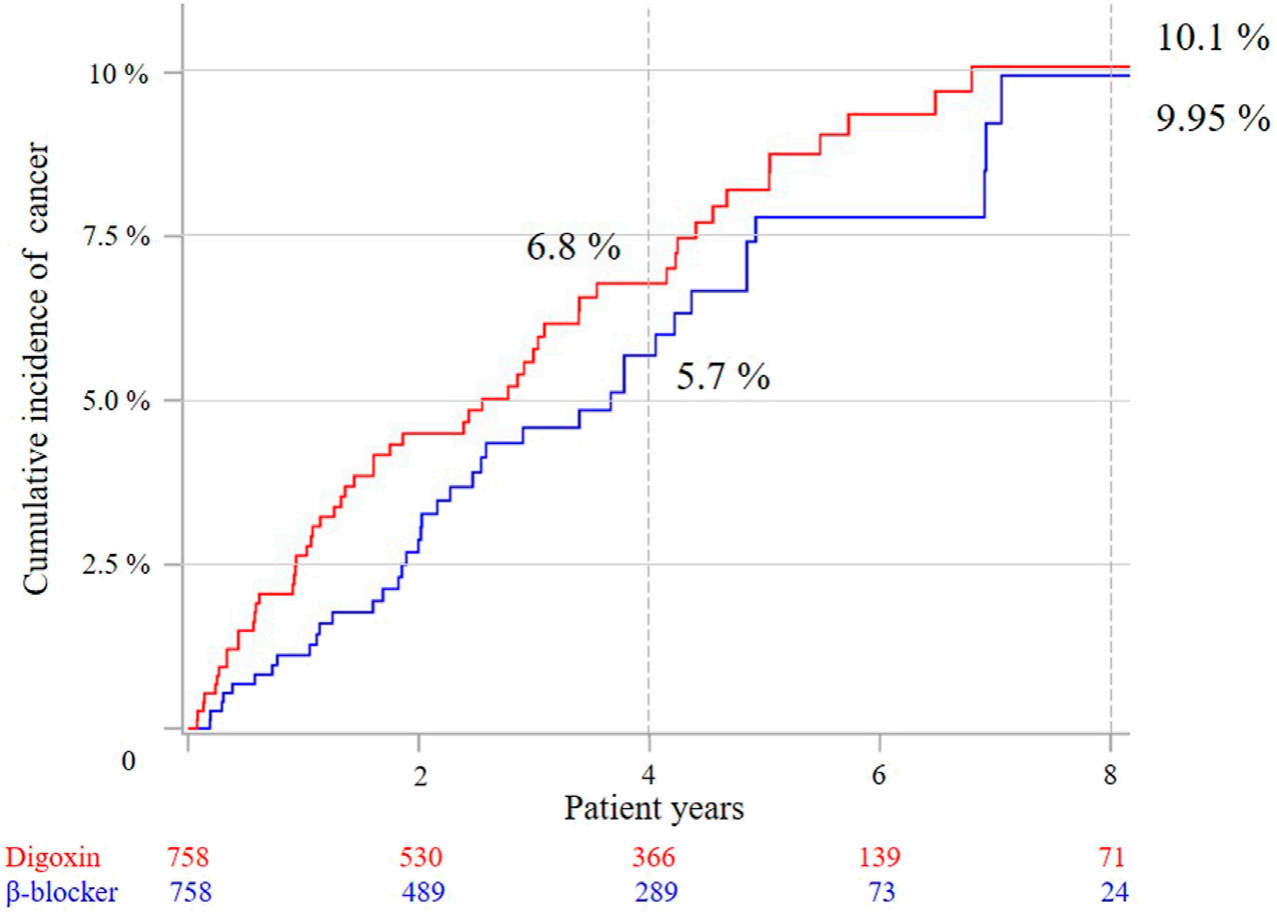
Cumulative cancer incidence between the two groups after 1:1 propensity-score matching by competing risk analysis.

After 1:1 propensity-score matching by covariates, the all-cause mortality was statistically higher for the digoxin group (10.4 vs. 4.9%) in the 4 years follow-up and 8 years follow-up (13.6 vs. 7.0%) (Table 2). Competing risk analyses indicated that the digoxin group had higher cancer cumulative incidence than the β-blocker group (Figure 3). Moreover, subdistribution HR (SHR) calculated by proportional subdistribution hazard regression was 1.99 for the digoxin group (95% CI: 1.22–3.01) in the 4- years follow-up, and 1.46 (95% CI: 1.22–3.01) in the 8 years follow-up (Table 2). The adjusted HR (adjHR) estimated by traditional Cox proportional regression model showed no statistical difference between the two groups.

**TABLE 2:** All-cause Mortality and Cancer incidence Between Two Groups After 1:1 propensity score-matching.

	Digoxin group *n* = 758	β-blocker group *n* = 758	Adjusted hazard ratio (95%CI)	*p* value	Subdistribution hazard ratio (95%CI)	*p* value
4-year follow-up						
All-cause mortality	79 (10.4%)	37 (4.9%)	1.74 (1.18–2.59)	0.006^a^	NA	NA
Cancer incidence	43 (5.7%)	30 (4.0%)	1.26 (0.79–2.02)	0.34	1.99 (1.22–3.01)	0.006^a^
8-year follow-up						
All-cause mortality	103 (13.6%)	53 (7.0%)	1.41 (1.01–1.97)	0.046^a^	NA	NA
Cancer incidence	57 (7.5%)	40 (5.3%)	1.17 (0.78–1.76)	0.45	1.46 (1.01–2.15)	0.054

CCB = calcium channel blocker; CI = confidence interval; Cox proportional hazards regression.

^a^ *p* value < 0.05Subdistribution hazards regression.

^b^ *p* value < 0.05.

## Discussion

Given that natural cardiac glycosides showed an anti-cancer property in a verity of cancer cells, but also because the discovering Na^+^/K^+^-ATPase can function as a signal transducer, the opportunity of repurposing digoxin as well as the development of other cardiac glycosides as anti-cancer drugs have increased interest in previous studies (Prassas and Diamandis, 2008). Digoxin exhibits anticancer property through inducing apoptotic and immunogenic cell death (Menger et al., 2012; Kulkarni et al., 2016). It also disturbed HIF-1, FAK, and NFκB pathways, which potential underlie its anti-angiogenic effect (Trenti et al., 2017; Wang et al., 2017; Ouyang et al., 2018). In contrast, the previous study suggested that an estrogen-like effect of digoxin might increase the incidence of breast cancer who receive hormone therapy (Biggar, 2012). Therefore, the anti-cancer properties of digoxin were inconclusive and different in specific cancer types. Besides, the narrow therapeutic window of digoxin leads to a consideration of drug impact on normal cells, which has not been evaluated in previous *in vitro* studies (Botelho et al., 2019). Concerning effectiveness and safety, the results of clinical observations appear to be of higher priority than preclinical studies.

To evaluate the association of digoxin and cancer incidence in clinical condition, we conducted a population-based, propensity score-matched, retrospective cohort study with competing risk analysis, which was not applied to previous observational studies. Moreover, we put concomitant medications into propensity-score matching covariates, which minimized the confounding by indication because the concordance between patients’ clinical condition with medication use was higher than concordance with clinical diagnosis (Wu et al., 2014). Our result showed that digoxin group had significantly higher cancer incidence and risk than the β-blocker group.

### Conventional Survival Analysis vs. Competing Risk Analysis

KM survival analysis and Cox proportional hazards regression are often adapted for assessing the time to an event such as death or drop-out from the cohort study (Hsieh et al., 2015). When these methods are used to describe outcomes other than all-cause mortality in patients with a significant risk of death, the result may be biased (Berry et al., 2010). The cancer incidence and adjHR calculated by Cox regression model showed no significant difference between the digoxin group and the β-blocker group. However, all-cause mortality was significantly different between the two groups in the current study. The subdistribution hazards regression demonstrated that death events might be treated as censored events, and the risk might be underestimated in the conventional Cox proportional regression (Lau et al., 2009). Therefore, the current study proposed that competing risk analysis should be commonly applied for similar clinical observation studies.

### Cancer Risk of Digoxin Use Was Influenced by Concomitant Medications

Digoxin users often took concomitant medications, such as aspirin, ACEI, and ARB because of complicated clinical conditions. However, these medications might influence cancer incidence. For example, clinical recommendations for aspirin-based chemoprevention strategies have recently been established for the prevention of cancer (Drew et al., 2016). However, the efficacy of clopidogrel or warfarin as an anticancer agent was inconclusive (Haaland et al., 2017; Bruno et al., 2018). Moreover, although recent meta-analysis studies showed neutral effects of ACEI/ARB on cancer incidence (Bangalore et al., 2011; Zhao et al., 2016), the association between cancer risk and ACEI/ARB still had conflicting conclusions. In contrast, amiodarone may be associated with an increased risk of incident cancer, especially in men (Su et al., 2013). Therefore, possible concomitant medications might interfere with the interpretation of the previous studies (Chung et al., 2017; Xie et al., 2017). The current study used propensity-score matching to minimize the effect of concomitant medications.

### The Association of Cancer Risk and Digoxin and Hormone Receptors

Ahern and colleagues observed an increased risk of breast cancer in Danish patients treated with digoxin (OR:1.30; 95% CI: 1.14–1.48) (Ahern et al., 2008). However, 32–37% of patients received hormone replacement therapy in that study and the study did not include subgroup analysis of breast cancer incidence in patients without hormone replacement therapy. The result might be interfered with the effect of hormone replacement therapy on the incidence of breast cancer. Ahern and colleagues also studied the Unites States registered nurses database, where the results indicated higher HR in digoxin users in the estrogen receptor (ER)-positive breast cancer (HR = 1.45; 95% CI: 1.13–1.86) compared to the ER-negative breast cancer (HR = 1.21; 95% CI: 0.52–2.37) (Ahern et al., 2014). However, data on digoxin exposure in that study was collected by questionnaires biennially, which might not reflect the true medical adherence and dose-response effects. In our opinion, physicians should pay more attention to the concomitant use of digoxin and hormone therapy.

Although cardiac glycosides decrease expression of prostate-specific antigen by down-regulation of prostate-derived Ets factor (Juang et al., 2010), digoxin treatment did not show conclusive anti-cancer effects on prostate cancer in previous conducted studies. Platz and colleagues suggested that regular digoxin users for ≥10 years had a lower prostate cancer relative risk of 0.54 (95% CI 0.37–0.79, p-trend <0.001) compared to nonusers (Platz et al., 2011). In contrast, Kaapu and colleagues showed no significant association was between digoxin use and overall prostate cancer risk (OR = 0.95; 95% CI: 0.89–1.01) or advanced prostate cancer risk (OR = 0.90; 95% CI: 0.77–1.05) (Kaapu et al., 2015). The report concluded that sotalol, but not digoxin was associated with decreased prostate cancer risk.

### Limitations

Although the study generated significant findings, our results should be interpreted with caution. Firstly, the results are limited to patients with HF, AF or AFL. The effect of digoxin on healthy participants could not be obtained in the NHIRD study. Secondly, although we used multiple strategies to minimize confounding by propensity-score matching, the current observational study might have residual confounding factors and cannot prove causality. Third, the medical adherence and some time-varying changes might influence the result. Therefore, the result was less evident in 8 years than 4 years follow up. Finally, the incidence of specific cancer types was not evaluated because the cancer case numbers were too low for advance analysis.

### Insights for Further Translational Study and Clinical Trials

Although digoxin proves its anti-cancer effects *in vitro*, for now, it is virtually impossible to perform a clinical trial to evaluate the association of digoxin and cancer incidence for the healthy population because of the safety issues of digoxin. We suggest that researchers can screen natural cardiac glycosides with the same or better anti-cancer effects but lower cardio-inotropic effects compared with digoxin.

According to previous studies, the possible anti-cancer mechanisms of β-blockers were as follows. The mode of action of β-blockers *in vitro* linked to cancer growth inhibition through impaired production of vascular endothelial growth factor (VEGF) (Watkins et al., 2015), decreased cytokine serum levels (Haldar et al., 2018), and enhanced the effect of epidermal growth factor receptor (EGFR) inhibitors (Nilsson et al., 2017). One observational study indicated that patients taking propranolol had an 80% risk reduction in melanoma recurrence (De Giorgi et al., 2018), while another study suggested that the use of β-blockers might be associated with more prolonged survival in patients with stage IV colorectal cancer (Jansen et al., 2014). However, anti-cancer effect dose not equal to the prevention of cancer. There is still a knowledge gap between anti-cancer effect derived from basic study and clinical evidence of cancer prevention. Further basic study and clinical trial are needed to fill the gap.

## Conclusion

Our study is the first to include the death event as the competing risk while evaluating the association of digoxin and cancer incidence. Our results suggest traditional Cox regression model underestimated the risk. After considering the risk of death, digoxin users had a higher risk of cancer incidence compared to β-blockers. The current study demonstrated the necessity of competing risk analysis applying to similar clinical researches.

For clinical use for cancer prevention, the assessment of digoxin or other natural derived cardiac glycosides should consider their safety issue first. Further studies are required to evaluate their effectiveness.

## Data Availability

The data analyzed in this study is subject to the following licenses/restrictions: The use of dataset needs the permission of the Ministry of Health and Welfare, Taiwan. Requests to access these datasets should be directed to Ministry of Health and Welfare, Taiwan.
